# Relationship between risk assessment and payment models in Swedish Public Dental Service: a prospective study

**DOI:** 10.1186/s12903-016-0327-4

**Published:** 2017-01-11

**Authors:** Gunnel Hänsel Petersson, Svante Twetman

**Affiliations:** 1 Department of Cariology, Faculty of Odontology, Malmö University, SE-205 06 Malmö, Sweden; 2 Department of Odontology, Faculty of Health and Medical Sciences, University of Copenhagen, Copenhagen, Denmark

**Keywords:** Caries prevention, Dental care models, Risk assessment, Young adults, Sweden

## Abstract

**Background:**

To a) compare risk categories in patients selecting a capitation payment (CP) model with those in fee-for-service (FFS), b) determine the 3-year caries increment in the two groups, and c) compare the amount of delivered preventive care in the two groups.

**Methods:**

A comprehensive risk assessment was carried out in 1295 young adults attending eight Public Dental Clinics in the Scania region and 75% could be re-examined after 3 years; 615 had selected the CP model and 310 the traditional FFS. Caries was scored according to WHO and data concerning preventive care was extracted from the dental records.

**Results:**

More patients in the low risk category preferred the CP model (74% vs. 26%) while >80% with high risk selected FFS. The baseline caries level was significantly higher in the FFS group as well as the 3-year caries increment (1.6 vs. 0.8 DFS: *p* < 0.05). The amount of additional preventive care delivered to each patient was generally lower in the FFS model; it was most frequent among patients with “some” risk in the CP model (83.8%) while the lowest delivery rates were found among low risk patients in the FFS system (32.4%).

**Conclusions:**

Young adults in public dental care with low risk preferred the prepaid model while those in the higher risk categories selected fee-for-service. As more additional preventive care was delivered to patients in the subscribed care, oral health planners and decision makers should be aware of the fact that capitation payment models may enhance inequalities in dental health over time.

## Background

Dental care in Sweden is provided by the Public Dental Service or by private practitioners free of charge for the patients younger than 20 years of age. The costs are covered by a National Dental Insurance through a capitation system constructed to promote health and encourage preventive care. At the age of 20 years, all patients that wish to remain in the Public Dental Service have the opportunity to choose one of two payment systems, a) a monthly fixed payment (capitation payment, CP) or, b) a traditional fee-for-service system (FFS) with payments for each treatment that is carried out. In the former payment model, the patient signs a 3-year contract with the clinic at a fixed fee covering all necessary basic dental care over the time period. A subscription agreement for dental care includes check-ups, preventive procedures, treatment of the disease and restorative care (fillings and single crowns). For example orthodontics, aesthetic dental care and implants are excluded from the basic care. The CP fee is based on a comprehensive risk assessment and the higher the risk, the higher the fee. The methodology for the risk assessment, as well as the size of the fee, varies however somewhat across different regions in Sweden and also over time. So does the share of patients over 19 years of age preferring the CP model, varying from a few percent in more rural areas to up to more than 50% subscribers in other regions.

Although the CP model was introduced more than 10 years ago, few studies have been published on its effect on oral health and whether or not this is beneficial for the patients and/or the organisation [1, 2]. There are some international low-quality evidence to suggest that the payment method does influence the behaviour of dentists in child dental care with a tendency to higher activity and more preventive advice in the capitation system [2]. Among adults, it has been shown that patients selecting the CP model had healthier habits and were more motivated to follow self-care advice than those adhering to FFS system [3]. As a consequence, fewer fillings were carried out among the patients in the pre-payment scheme [4]. Furthermore, the CP patients received more emergency and preventive treatments and were more frequently examined by dental hygienists than the FFS patients [5].

We have previously validated a risk assessment model in a population of young adults living in the Scania region in southern Sweden over a period of 3 years [6]. After the assessment, the patients had to choose the CP model for the next 3 years. Those who not selected the CP model were offered continues care based on FFS. This gave us an opportunity for a pragmatic comparison of the two payment models with respect to caries risk and caries development. The aims of the study were a) to compare risk categories in patients selecting the CP or FFS model, b) to determine the 3-year caries increment in the two groups, and c) to compare the amount of delivered preventive care in the two groups.

## Methods

**Fig. 1:**
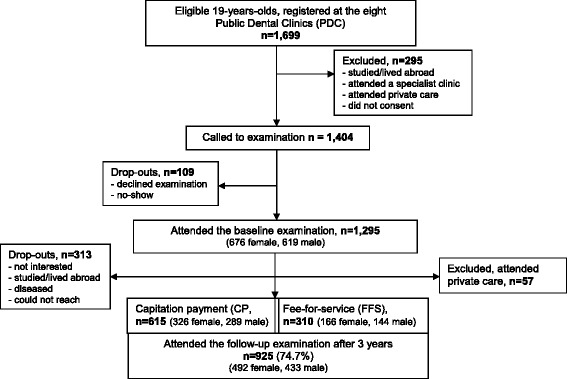
Flow-chart indicating attrition and reason for drop-outs

The selection and enrolment of the study cohort has been described before [6]. In brief, 1699 19-year-olds were invited from eight different Public Dental Clinics (PDC’s) across the region to form a representative sample of this age group. At baseline, 1295 patients were examined and risk assessed according to the regional guidelines. After 3 years, 925 patients who remained at the PDC could be re-examined; 615 with a CP payment scheme and 310 with FFS payments. A flowchart with the sex distribution and reasons for exclusion and drop out is provided in Fig. 1. All the participants were residents in areas with low natural fluoride content in the drinking water supply but were strongly encouraged to use of fluoridated dentifrice on daily basis. The study design was approved by the Ethical Committee, Lund University, Sweden.

### Study design and risk assessment

The study had a 3-year prospective observational design. At baseline, the risk category of each patient was assessed by the patient’s regular dental team (dentist and/or dental hygienist) according to the “adult guidelines for risk assessment of oral diseases” issued by the regional Public Dental Service. Based on clinical and radiographic findings, the risk grouping relied on past and present caries, periodontal conditions, general risk and technical risk as previously described [6]. Four risk categories were used and each patient was classified into one of the following risk groups; “low risk” (42.7%), “some risk” (45.4%), “high risk” (10.4%) and “very high risk” (1.5%). The patient was informed on the outcome of risk assessment and asked to choose payment model. All decisions on preventive and restorative dental care were solely the responsibility of the patient’s regular dental team during the entire study period. All patients, irrespective of payment model, were regularly attending the Public Dental Service for check-ups.

### Clinical examination

Prior to baseline, each of the eight selected public dental clinics was visited by the principal investigator (GHP). The staff was trained and calibrated on caries detection and different stages of lesions were discussed. The clinical visual-tactile examination, including bitewing radiographs, was carried out by the regular dentist, or a dental hygienist, under optimal light and cleaned, air-dried teeth. Caries prevalence and experience was registered at manifest dentin level according to the WHO-criteria [7] and expressed as DFT/DFS. Information concerning general health and medication, diet and oral hygiene habits including tooth brushing frequency and the use of fluoride and tobacco was collected through a structured questionnaire and these data were incorporated in the risk assessment model. The caries increment was calculated by counting the number of teeth/surfaces that changed from sound to decayed or filled over the study period. Possible caries reversals were not considered.

### Preventive measures

Data on the delivered preventive care to each patient over the 3-year study period was extracted from the digital dental records by one of the authors as previously described [8]. In brief, the frequency of activities within three domains was scored 0–3: “oral health information” (i.e. current disease situation, diet information and counselling), “extra fluoride therapy” (i.e. fluoride varnish application, additional fluoride supplements), and “professional tooth cleaning and oral hygiene instructions”. The scores of the three domains were then added to reflect the total preventive care delivered to each patient with a maximal cumulative score of nine. A cumulative score between 0 and 3 was regarded as “basic prevention” while 4–9 was considered as “additional prevention”.

### Statistical methods

All data were processed with the IBM-SPSS software (version 23.0, Chicago, IL, USA). Descriptive statistics were employed and differences between the groups and were compared with chi-square test for proportions and non-parametric two-tailed tests for continuous data. P-values less than 0.05 were considered statistically significant.

## Results

**Fig. 2:**
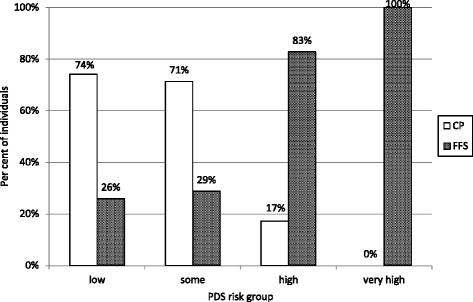
Distribution of individuals (%) in the different Public Dental Service risk categories at baseline in the capitation payment group (CP) and fee-for-service group (FFS)

**Fig. 3:**
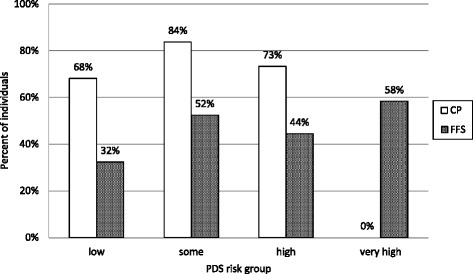
Percentage received “additional preventive care” in the capitation payment group (CP) and the fee-for-service group (FFS), in relation to the baseline risk categories

At follow-up there were an equal proportion of females in the CP and FFS groups, 53.0 and 53.5% respectively. The distribution of the risk categories at baseline is shown in Fig. 2. Significantly more patients in the “low” or “some” risk categories selected the prepaid model while all those assessed with “very high” risk preferred the FFS system (*p* < 0.05). The mean caries frequency at baseline and the 3-year caries increment is shown in Table 1. The patients in the FFS group had significantly more caries at baseline compared with the CP group (*p* < 0.05) and the mean 3-year increment was also significantly higher, 1.6 vs. 0.8 DFS. The additional delivered preventive care in the two groups in relation to the baseline risk category is shown in Fig. 3. The proportion of patients that received “additional preventive care” was significantly higher in the CP group (*p* < 0.05). Additional prevention was most frequent in patients with “some” risk in the CP model (83.8%). The lowest frequency was seen in the low risk FFS group (32.4%).

**Table 1:** Mean (SD) caries prevalence at baseline and mean (SD) caries prevalence and increment after 3 years in the CP (*n* = 615) and FFS (*n* = 310) groups

Group	DFT	DFS	DFSa
CP - baseline	2.74 (2.70)	3.64 (4.16)	0.70 (1.59)
CP - after 3 years	3.23 (3.05)	4.44 (4.85)	0.98 (2.00)
CP - increment	0.48 (0.90)	0.77 (1.53)	0.29 (0.74)
FFS – baseline	3.75 (3.55)	5.39 (6.05)	1.41 (2.65)
FFS - after 3 years	4.55 (4.05)	6.96 (7.54)	2.19 (3.57)
FFS - increment	0.80 (1.33)	1.57 (2.69)	0.78 (1.54)

*CP* capitation payment, *FFS* fee-for-service, *DFT* Decayed Filled Teeth, *DFS* Decayed Filled Surfaces, *DFSa* Decayed Filled approximal Surfaces

## Discussion

In this material of young adults, 66.5% utilized the capitation model at the 3-year follow-up and this proportion was substantially higher than the national average. However, according Andrén Andås and Hakeberg [3] and a national social insurance report [9], young adults (20–29 years) were clearly overrepresented among subscription dental care patients and they were more often females. Interestingly, this gender difference was not unveiled in our present material. One should however keep in mind that there was a possible risk for selection bias since 28.5% of the patients examined at baseline was lost to follow-up. It seemed to be no apparent reason for dropping out although lack of interest and migration were most commonly registered. Interestingly, only 4.4% of the originally enrolled subjects had moved to private practitioners after 3 years. Although data on caries frequency was available, the private patients were excluded due to lack of information concerning the delivered preventive care. Furthermore, patient’s attending private care did not have the opportunity to select the capitation model since this is not available in private dental care.

The main finding of this study was that young adults assessed with low risk for caries development, periodontal disease or technical problems, were more likely to adopt the capitation model. Over 70% in the “low” and “some” risk groups preferred to pay a fixed rate and it seemed clear that the subscription dental care largely attracted those with good oral health and presumably, small dental care needs. Only 17% of those assessed with high risk, and thereby likely to have more extensive treatment needs and unforeseen costs, signed the agreement. No one in the very high risk group selected the CP model, which was notable. An explanation for this could be that the annual fee was seven times higher in the very high risk group compared to the lowest risk category, indicating that the economical factor had a pivoting influence the patients’ choice of payment model. Our findings were therefore basically in support of the observations made by Andren Andås and Hakeberg [3] who suggested that patients choosing prepaid care were young, educated people that judged their oral health to be good or very good.

As could be expected from the distribution of the risk categories, the patients in the FFS group had more caries at baseline and displayed a significantly higher DFS increment over the 3-year study period. Similar observations have been made in children [10] and in adults [5]. The latter research team showed also that subscription patients received significantly more prevention and fewer fillings than FFS patients. We were however unable to fully disseminate the reasons for this difference in caries burden in our material. First of all, the patients in the CP group received more additional prevention than those selecting the FFS model. A paradox was that more prevention actually was delivered to the “low risk” and “some risk” groups than the high risk group over a 3-year period [8]. This is however not a unique observation from dentistry but well-known from general health care as the “inverse care law” [11]. The primary method of choice was professional fluorides which are in systematic reviews proven to be effective for both primary and secondary prevention [12, 13]. A second reason could be that dentists in the CP model restored lesions at later stages [14]. Subscription patients are more likely to return on a regular basis [9] and this fact can promote a tendency towards watchful waiting and more preventive care. In a wider perspective, this is an obvious ethical dilemma. The overarching goal of subscription dental care is to encourage regular preventive-oriented measures and to retain customer loyalty. The economical outcome seems also positive; a recent study has shown that the revenue from the prepaid fees is exceeding the costs for the provided care over time [5]. There is however an obvious risk that the inequality gaps in caries burden across the population will become wider with time. The occurrence of caries has a strong socioeconomic gradient in all age groups [15, 16] and the disadvantaged socioeconomic groups are generally found in the FFS system [17]. Moreover, displacement effects cannot be excluded if low risk subscription patients are crowding out patients with more extensive needs. Theses aspects must be considered and addressed by regional chief dental officers and oral health planners.

In this study, we did not have the possibility to investigate the patient’s perspective on the different payment systems. Previous reports indicate however that patients generally were in favour of the capitation payment system [18] and that the individual’s relation to the public dental clinics together with his/her health-related attitudes and perceptions were the main factors impacting the choice of payment system [19]. In addition, Johansson and co-workers have shown that patients in prepaid care had better general health [17] and a better oral health related quality in life than those in FFS [20]. Thus, it is a future challenge to communicate the benefits of subscribed prepaid care also to patients with high risk and increased treatment needs. With an enhanced preventive approach, individual patients may lower their risk category and spread the costs for dental care out over time.

## Conclusions

Within the limitations of the present study, our findings displayed that young adults in public dental care assessed with low risk preferred a prepaid capitation model while those with higher risk categories selected the traditional fee-for-service. The mean caries increment among the fee-for-service patients were significantly higher compared with the prepaid group over a 3-year period and more additional preventive care was delivered to patients in the subscribed group. Thus, oral health planners and decision makers should be aware of, and deal with, the fact that capitation care may increase inequalities in dental health over time.

## Abbreviations

CP: Capitation payment
DFS: Decayed filled surfaces
DFSa: Decayed filled approximal surfaces
DFT: Decayed filled teeth
FFS: Fee-for-service
PDC: Public dental clinic
WHO: World Health Organisation
